# New Insights and Potential Therapeutic Interventions in Metabolic Diseases

**DOI:** 10.3390/ijms241310672

**Published:** 2023-06-26

**Authors:** Vicente Javier Clemente-Suárez, Alexandra Martín-Rodríguez, Laura Redondo-Flórez, Clara López-Mora, Rodrigo Yáñez-Sepúlveda, José Francisco Tornero-Aguilera

**Affiliations:** 1 Faculty of Sports Sciences, Universidad Europea de Madrid, Tajo Street, s/n, 28670 Madrid, Spain; 2 Grupo de Investigación en Cultura, Educación y Sociedad, Universidad de la Costa, Barranquilla 080002, Colombia; 3 Department of Health Sciences, Faculty of Biomedical and Health Sciences, Universidad Europea de Madrid, Tajo Street s/n, 28670 Villaviciosa de Odon, Spain; 4 Facultad de Ciencias Biomédicas y de la Salud, Universidad Europea de Valencia, Pg. de l’Albereda, 7, 46010 València, Spain; 5 Faculty of Education and Social Sciences, Universidad Andres Bello, Viña del Mar 2520000, Chile

**Keywords:** genetic, psychology, single-cell transcriptomics, gut, epigenetics, advanced imaging techniques, cell-based therapies, microbiota, nutrition, physical activity

## Abstract

Endocrine homeostasis and metabolic diseases have been the subject of extensive research in recent years. The development of new techniques and insights has led to a deeper understanding of the mechanisms underlying these conditions and opened up new avenues for diagnosis and treatment. In this review, we discussed the rise of metabolic diseases, especially in Western countries, the genetical, psychological, and behavioral basis of metabolic diseases, the role of nutrition and physical activity in the development of metabolic diseases, the role of single-cell transcriptomics, gut microbiota, epigenetics, advanced imaging techniques, and cell-based therapies in metabolic diseases. Finally, practical applications derived from this information are made.

## 1. Introduction

Metabolic diseases are a group of disorders characterized by abnormal metabolism, which refers to the chemical reactions that occur within cells to convert food into energy and other essential molecules. Nowadays, going through each of them, changes in the incidence of obesity, diabetes mellitus, nonalcoholic fatty liver disease, and their associated hyperlipidemia, the contribution of genetics, and the function of the endocrine system in these metabolic disorders are discussed [1]. The global obesity epidemic affects infants, adolescents, and adults. In the past three decades, the global prevalence of obesity has nearly doubled. For the first time on a global scale, the number of obese and overweight individuals outnumbers those who are underweight [1]. Also, diabetes mellitus (DM) is a group of metabolic diseases characterized by hyperglycemia resulting from defects in insulin secretion, insulin action, or both. DM can result from a deterioration in function and/or a loss of mass in pancreatic tissue [2]. Also, the liver is the primary organ involved in lipid metabolism. Some consider nonalcoholic fatty liver disease (NAFLD) [3], which is characterized by excessive triglyceride accumulation within hepatocytes (steatosis), to be the hepatic manifestation of obesity and metabolic syndrome (MetS) [4]. NAFLD is the most prevalent liver disease, affecting 25% of the world’s population. Finally, MetS is defined as a cluster of the most hazardous risk factors for heart attack, including diabetes and prediabetes, abdominal obesity, high cholesterol, and high blood pressure [1].

It is well known that regular exercise can benefit health by enhancing antioxidant defenses in the body. However, unaccustomed and/or exhaustive exercise can generate excessive reactive oxygen species (ROS), leading to oxidative stress-related tissue damage and impaired muscle contractility. ROS are produced in both aerobic and anaerobic exercise. Mitochondria, NADPH oxidases, and xanthine oxidases have all been identified as potential contributors to ROS production, yet the exact redox mechanisms underlying exercise-induced oxidative stress remain elusive. Interestingly, moderate exposure to ROS is necessary to induce the body’s adaptive responses, such as the activation of antioxidant defense mechanisms. Dietary antioxidant manipulation can also reduce ROS levels and muscle fatigue, as well as enhance exercise recovery. To elucidate the complex role of ROS in exercise, this review updates on new findings of ROS origins within skeletal muscles associated with various types of exercises such as endurance, sprinting, and mountain climbing. In addition, we will examine the corresponding antioxidant defense systems as well as dietary manipulation against damages caused by ROS [5]. The most common metabolic diseases include obesity, type 2 diabetes, and cardiovascular disease, which are characterized by dysregulation of glucose and lipid metabolism. In these conditions, the body is unable to properly use and store energy from food, resulting in elevated blood glucose and lipid levels. Metabolic diseases can result from a combination of genetic, environmental, and lifestyle factors, such as a high-calorie diet, physical inactivity, and genetic predisposition [6]. Left untreated, metabolic diseases can lead to serious complications, such as heart disease, kidney disease, and nerve damage. Understanding, from a holistic point of view, the underlying molecular mechanisms involved in metabolic diseases is critical for the development of effective prevention and treatment strategies. In this line, new insights into metabolic diseases have emerged in recent years, revealing the complex interplay of genetic, environmental, and lifestyle factors that contribute to their development.

Genetic studies have identified numerous loci associated with metabolic diseases, shedding light on the underlying molecular pathways involved. Metabolic diseases encompass a broad range of disorders affecting the body’s ability to regulate and utilize energy, including type 2 diabetes, obesity, and cardiovascular disease [7]. Through genome-wide association studies (GWAS), researchers have identified genetic variants linked to these diseases, shedding light on the underlying biological mechanisms involved. Additionally, research has revealed that epigenetic modifications, such as DNA methylation and histone modification, play a critical role in regulating gene expression and contributing to metabolic disease risk. Studies have also shown that environmental factors, such as diet and physical activity, can modulate the epigenetic marks associated with metabolic disease risk [8]. For example, a high-fat diet can alter DNA methylation patterns in metabolic tissues, potentially contributing to the development of metabolic diseases [9]. These findings have not only deepened our understanding of metabolic diseases but have also paved the way for the development of novel therapies and preventative measures. As genetic research continues to advance, we can hope to gain further insights into the intricate genetic architecture underlying metabolic diseases, which will ultimately lead to improved diagnosis, treatment, and prevention strategies.

Additionally, psychological factors such as depression, anxiety, and chronic stress have been shown to contribute to the development of metabolic diseases through their effects on hormonal and metabolic pathways [10]. For example, chronic stress has been linked to increased cortisol levels, which can lead to insulin resistance and impaired glucose metabolism, thus leading to metabolic disease. Furthermore, behavioral factors, including sleep quality, sedentary behavior, and smoking, have also been shown to contribute to the development of metabolic diseases [11]. Poor sleep quality and duration have been linked to an increased risk of obesity and type 2 diabetes, potentially due to their effects on appetite regulation and insulin sensitivity. Sedentary behavior, such as sitting for prolonged periods, has also been associated with an increased risk of metabolic diseases, even in individuals who engage in regular physical activity [12]. Additionally, smoking has been linked to increased insulin resistance and a higher risk of type 2 diabetes. Finally, another cornerstone, nutrition, is postulated as another critical factor in the development and management of metabolic diseases. A diet high in refined carbohydrates, saturated and trans fats, and added sugars has been linked to an increased risk of metabolic diseases [13]. These types of diets can contribute to insulin resistance and dyslipidemia, both of which are important risk factors for metabolic diseases. On the other hand, a diet rich in whole grains, fruits, vegetables, and lean protein has been associated with a lower risk of metabolic diseases [14]. These types of diets can improve insulin sensitivity, reduce inflammation, and lower the risk of obesity and type 2 diabetes. A recent review suggests that it is not only the types of foods consumed but also the timing of meals and overall dietary patterns that play a role in metabolic health outcomes [15]. In this line of research, intermittent fasting and time-restricted eating have been shown to improve glucose metabolism and insulin sensitivity in individuals with metabolic diseases. Additionally, dietary interventions, such as calorie restriction and low-carbohydrate diets, have been shown to improve metabolic health and glycemic control in individuals with type 2 diabetes [16].

Overall, the relationship between psychological and behavioral factors, nutrition, and metabolic diseases highlights the importance of a comprehensive approach to disease prevention and management. Interventions aimed at improving psychological well-being, such as stress management, cognitive behavioral therapy, and the implementation of appropriate dietary patterns, may have a key role in preventing or managing metabolic diseases.

Regarding new approaches, single-cell transcriptomics, a powerful tool for studying the molecular mechanisms underlying metabolic diseases, is recently getting a lot of attention. This technology allows for the profiling of gene expression in individual cells, providing insights into the cellular heterogeneity and functional diversity within tissues that are not detectable with traditional bulk transcriptomic approaches. In this vein, recent studies have used single-cell transcriptomics to identify novel cell types and pathways that contribute to metabolic diseases [17]. For example, studies have identified new subpopulations of adipose tissue cells that are involved in the regulation of thermogenesis, inflammation, and lipid metabolism [18]. Other studies have used single-cell transcriptomics to identify new targets for drug development, such as cell surface markers that are selectively expressed on adipose tissue macrophages in obesity [19]. As such, single-cell transcriptomics has also been used to study the effects of environmental and genetic factors on cellular function in metabolic diseases. For example, a study of human pancreatic islet cells found that exposure to a high-fat diet altered the expression of genes involved in insulin secretion and glucose metabolism [20].

From these advances, new promising techniques such as cell-based therapies are emerging as a promising approach for the treatment of metabolic diseases. These therapies involve the transplantation of cells, either from a donor or generated through in vitro differentiation of stem cells, to replace or augment the function of damaged or dysfunctional cells in the body [21]. However, the success of these therapies has been limited by the need for immunosuppressive drugs to prevent the rejection of the transplanted cells as well as the shortage of donor islet cells. Therefore, further research is needed. However, with the advances in imaging techniques such as magnetic resonance imaging (MRI) and positron emission tomography (PET), the advances and speed in metabolic disease research are promising. For example, studies have used MRI to examine changes in brain structure and function in individuals with type 2 diabetes and obesity, identifying alterations in brain regions involved in appetite regulation and reward processing [22]. MRI has also been used to study the effects of dietary interventions and bariatric surgery on the liver and adipose tissue, providing insights into the mechanisms underlying improvements in insulin sensitivity and metabolic function [23]. As for PET, studies have used it to measure glucose uptake in the brain, liver, and skeletal muscle, providing insights into the changes in glucose metabolism that occur in these tissues in conditions such as type 2 diabetes and obesity [24]. PET has also been used to investigate the effects of exercise and other interventions on metabolic activity in these tissues [25]. Yet other advanced imaging techniques, such as computed tomography (CT) and optical imaging, have also been used to study metabolic diseases. CT has been used to investigate changes in bone mineral density and body composition in conditions such as osteoporosis and obesity [26]. Overall, advanced imaging techniques have provided important insights into the structural and functional changes that occur in tissues and organs affected by metabolic diseases. These techniques may lead to the development of new diagnostic tools and the identification of new therapeutic targets for these conditions.

In conclusion, this narrative review highlights the multifaceted nature of metabolic diseases and the importance of a comprehensive approach to their prevention and treatment. Incorporating recent advancements in genetics, psychology, movement, nutrition, single-cell transcriptomics, gut microbiota, epigenetics, advanced imaging techniques, and cell-based therapies will pave the way for new strategies to combat metabolic diseases.

## 2. Methods

In order to conduct this study, we employed an extensive literature search utilizing primary and secondary sources. Our search encompassed scientific articles, bibliographic indexes, and databases, including PubMed, Scopus, Embase, Science Direct, Sports Discuss, ResearchGate, and the Web of Science. To ensure the relevance of the literature, we utilized MeSH-compliant keywords such as diet, metabolic disease and genetics, epigenetics, psychology, exercise, nutrition, single-cell transcriptomics, gut microbiota, advanced imaging techniques, and cell-based therapies. To ensure the currency and pertinence of the data gathered, we focused on articles published from 1 May 2003, to 1 May 2023.

In order to ensure the appropriateness of the studies included in our analysis, a team of five review authors thoroughly examined the titles and abstracts of all retrieved manuscripts. We applied exclusion criteria to filter out studies utilizing outdated data outside the designated timeframe, studies with unrelated topics that did not align with the specific objectives of our study, and studies not written in English. Following the identification of relevant studies, the same team of review authors independently extracted information from the selected articles. Subsequently, we engaged in collaborative discussions to synthesize the findings and present the current narrative review. Our review encompasses a comprehensive analysis of the existing literature pertaining to the selected topics, thereby ensuring the reliability and currency of the data we present following previous research methodologies [27,28,29].

## 3. The Rise of Metabolic Diseases

In recent years, a concerning increase in metabolic disorders has been observed worldwide [30,31,32]. These disorders are associated with disorders or dysfunctions in the set of chemical and biochemical processes that take place in the body [29]. These processes aim to convert food into energy and, thus, carry out various vital functions, affecting the balance of chemicals in the human body, such as hormones, enzymes, and other key molecules for proper bodily function [33,34].

This group of diseases has become a significant burden on both individual and public health. This is evident in the data associated with the increased prevalence of these diseases, such as obesity and overweight, in both adult and child populations [35,36,37], and it is estimated to have a 3% impact on the global GDP by 2035 [38]. Diabetes, which affects between 5% and 20% of the population [39,40,41], and metabolic syndrome [42,43,44] are examples of these diseases. While metabolic diseases in some cases have a genetic origin [45], they can also be caused by environmental factors such as diet and sedentary lifestyles [25], high levels of stress [46,47,48], and/or disrupted sleep patterns [49,50].

In this regard, the literature indicates that it is important to pay attention to the phylogenetic processes undergone in the human species, which involve very little genomic evolution compared to the evolution of the current environment in which humans inhabit [29,51]. Although, since the Paleolithic era, humans have adapted their dietary behavior (greater food variety, food processing techniques, etc.) to cope with the high demands of the environment [51], since the establishment of intensive agriculture/animal husbandry and the industrial revolution, human nutritional behaviors have been modified (increased consumption of foods such as cereals and sugars), while energy expenditure demands have been reduced (more sedentary lifestyles), with minimal genetic adaptation. In other words, the rapid evolution and change in the current living environment, combined with negligible genetic modification, have resulted in evolutionary discordance [29], which has serious health consequences [29,51].

On the other hand, chronic stress, both physical and psychological, can have a negative impact on metabolism and trigger hormonal responses that affect metabolic balance [46,47,48]. Chronic stress has been associated with changes in food intake, a greater preference for high-fat and high-sugar foods, as well as a decrease in physical activity, which can increase the risk of metabolic diseases [51,52]. Another factor, such as disruption in sleep cycles, has also been linked to a higher risk of metabolic diseases [49,50,51,52,53,54]. Chronic sleep deprivation can lead to hormonal dysregulation and, thus, affect insulin sensitivity [55], contributing to the development of metabolic disorders such as type 2 diabetes and obesity.

In summary, the risk of developing metabolic diseases in the population is due to a combination of genetic, environmental, and lifestyle factors. Changes in diet, sedentary behavior, chronic stress, inadequate sleep, and exposure to toxic chemicals are factors that contribute to this concerning trend. To effectively address this problem, it is necessary to promote healthy lifestyles, educate about the importance of balanced nutrition, encourage regular physical activity, and minimize exposure to harmful chemicals.

## 4. Genetic Basis

Metabolic diseases can be broadly classified into two categories, those that result from genetic mutations and those that are acquired because of lifestyle factors such as diet and physical activity [56]. In the case of genetic metabolic diseases, mutations in specific genes can disrupt the normal functioning of metabolic pathways, leading to the accumulation of toxic metabolites or the depletion of essential metabolites. The specific genes involved in metabolic diseases vary depending on the disease in question, but there are several well-known examples that illustrate the genetic basis of these disorders [57].

### 4.1. Genetic Basis of Phenylketonuria and Cystic Fibrosis

**Figure 1:**
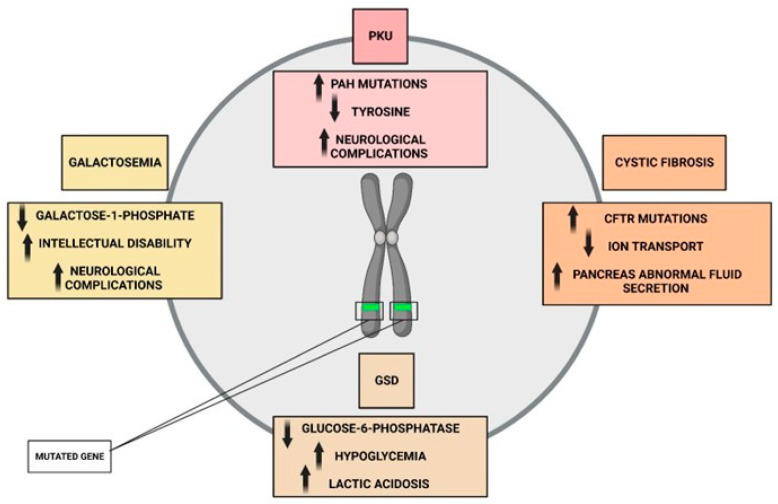
Representation of genetic mutations and their metabolic impairments.

One of the most well-known genetic metabolic diseases is phenylketonuria (PKU), which results from mutations in the gene encoding phenylalanine hydroxylase (PAH) [58]. PAH is an enzyme that converts the amino acid phenylalanine into tyrosine, and mutations in this gene lead to a build-up of phenylalanine and a deficiency of tyrosine. This, in turn, can lead to intellectual disability and other neurological problems if left untreated (Figure 1) [59].

Another example of a genetic metabolic disease is cystic fibrosis (CF), which is caused by mutations in the cystic fibrosis transmembrane conductance regulator (CFTR) gene (Figure 1) [60]. CFTR is a chloride channel that is involved in regulating ion transport across cell membranes, and mutations in this gene lead to abnormal fluid secretion in the lungs, pancreas, and other organs. This, in turn, leads to chronic lung infections, malabsorption of nutrients, and other complications [61]. In addition to PKU and CF, there are numerous other genetic metabolic diseases that have been identified, including galactosemia, glycogen storage diseases, and mitochondrial disorders. These diseases all result from mutations in specific genes that disrupt normal metabolic pathways, leading to a wide range of clinical symptoms. In other cases, metabolic diseases are caused by multiple genetic factors, each of which contributes to the overall risk of developing the disease. For example, type 2 diabetes is a complex disorder that is influenced by a combination of genetic and environmental factors. Genome-wide association studies have identified dozens of genetic loci that are associated with an increased risk of type 2 diabetes, many of which are involved in insulin secretion and glucose metabolism [62].

### 4.2. Common Mechanism in Genetic Mutations

The specific mechanisms by which genetic mutations lead to metabolic diseases vary depending on the disease in question. However, there are several common themes that can be observed across many different diseases. One common mechanism is the accumulation of toxic metabolites that are normally eliminated from the body. In the case of PKU, for example, the buildup of phenylalanine can lead to the production of toxic byproducts that damage brain tissue and lead to intellectual disability [59]. Another common mechanism is the depletion of essential metabolites that are required for normal cellular function. In the case of CF, for example, mutations in the CFTR gene lead to abnormal fluid secretion in the lungs, which can result in a depletion of mucus-clearing substances and an increased risk of chronic lung infections [61]. In addition to these mechanisms, there is growing evidence that metabolic diseases may be caused, at least in part, by dysregulation of the gut microbiome. Studies have shown that alterations in the composition of the gut microbiome can lead to changes in the metabolism of nutrients, and these changes can in turn contribute to the development of metabolic diseases such as obesity, type 2 diabetes, and non-alcoholic fatty liver disease [63].

### 4.3. Therapies against Molecular Mechanism

As our understanding of the genetic basis of metabolic diseases continues to grow, there is increasing potential for the development of targeted therapies that can address the underlying molecular mechanisms of these disorders. In some cases, these therapies may involve the replacement or supplementation of deficient enzymes or metabolites. For example, in the case of PKU, dietary supplementation with tyrosine and a low-phenylalanine diet can help prevent the buildup of toxic metabolites and reduce the risk of neurological complications [59]. In other cases, targeted therapies may involve the use of gene therapy to correct the underlying genetic mutations that cause metabolic diseases. While gene therapy is still in its early stages of development, there have been promising results in preclinical studies for a variety of genetic metabolic diseases, including CF and glycogen storage diseases [64]. There is also growing interest in the use of dietary interventions and lifestyle modifications to prevent or treat metabolic diseases. While these interventions may not directly address the underlying genetic mutations that cause these diseases, they can help modulate the metabolic pathways that are affected by these mutations and may provide an effective adjunct to other forms of therapy [65].

While genetic factors play an important role in the development of metabolic diseases, environmental factors such as diet and physical activity also play a critical role. In particular, the modern Western diet, which is high in refined carbohydrates and saturated fats, has been linked to the development of obesity, type 2 diabetes, and other metabolic disorders [65]. This is thought to be due, in part, to the impact of these dietary components on insulin sensitivity, inflammation, and oxidative stress. Physical activity is another important environmental factor that can influence metabolic health. Regular physical activity has been shown to improve insulin sensitivity, reduce inflammation, and promote the development of metabolically active muscle tissue [62]. Conversely, sedentary behavior has been linked to an increased risk of obesity, type 2 diabetes, and other metabolic disorders.

As our understanding of the genetic basis of metabolic diseases continues to grow, there is increasing potential for the development of targeted therapies that can address the underlying molecular mechanisms of these disorders. In some cases, these therapies may involve the replacement or supplementation of deficient enzymes or metabolites. For example, in the case of PKU, dietary supplementation with tyrosine and a low-phenylalanine diet can help prevent the buildup of toxic metabolites and reduce the risk of neurological complications [59]. In other cases, targeted therapies may involve the use of gene therapy to correct the underlying genetic mutations that cause metabolic diseases. While gene therapy is still in its early stages of development, there have been promising results in preclinical studies for a variety of genetic metabolic diseases, including CF and glycogen storage diseases [66]. Finally, there is also growing interest in the use of dietary interventions and lifestyle modifications to prevent or treat metabolic diseases. While these interventions may not directly target the underlying genetic mutations, they can modulate the expression of genes and pathways involved in metabolism, thereby improving metabolic health. For example, studies have shown that adherence to a Mediterranean-style diet, which is high in fruits, vegetables, whole grains, and healthy fats, is associated with a reduced risk of type 2 diabetes and other metabolic disorders [67].

**Figure 2:**
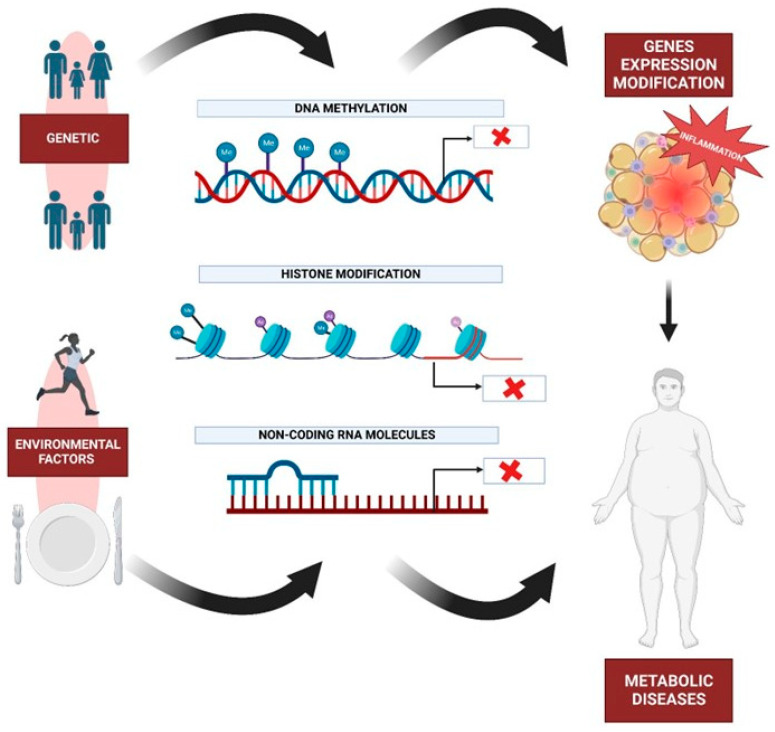
Graphic representation of genetic and epigenetic factors and their effects on metabolism. X codification.

In summary, metabolic diseases are a complex and heterogeneous group of disorders that are caused by a combination of genetic and environmental factors (Figure 2). While some metabolic diseases are caused by mutations in a single gene, others are caused by multiple genetic factors or environmental factors such as diet and physical activity. As our understanding of the genetic and molecular mechanisms underlying metabolic diseases continues to grow, there is increasing potential for the development of targeted therapies that can address these disorders at the molecular level. In addition, lifestyle modifications such as dietary interventions and regular physical activity may also play an important role in the prevention and treatment of metabolic diseases.

## 5. Psychological and Behavioral Basis

The metabolic diseases analyzed in this study are ultimately a result of altered eating behavior [68,69]. Eating behavior is a complex phenomenon that involves biological factors, such as reward mechanisms in the brain [70,71], taste mechanisms [71,72,73], as well as psychological factors, such as learning, motivation, or experiential avoidance [71,73,74,75].

From a transdiagnostic perspective of diseases, it is suggested that experiential avoidance is a phenomenon common to different diseases typified by the biomedical disease model [76]. In the process of understanding metabolic diseases from a transdiagnostic perspective, it is necessary to consider the role of psychological variables, with experiential avoidance being one that has gained more interest in recent years [77].

### 5.1. Experiential Avoidance and Metabolic Diseases

According to Hayes et al. (1996), experiential avoidance involves those elements present in the individual that led them to generate a recurring pattern of change or avoidance of discomforting private events (experiences, thoughts, memories, etc.) through their behavior, which, although it provides immediate relief from distress, is also associated with undesirable long-term outcomes [78].

The process of experiential avoidance is applicable to multiple contexts where individuals develop dysfunctional behavior in their environment, including metabolic diseases derived from unhealthy dietary patterns. It is worth noting that, in these diseases, the nutritional function of food takes a backseat as it is used as a self-management tool in situations perceived as threatening, stressful, or aversive by the individual [79,80]. In the pursuit of immediate pleasure (facilitated through food) driven by the need to avoid internal aversive experiences, hyperpalatable foods (highly processed foods) become a quick pathway for avoiding unpleasant private events.

This is evidenced by the study conducted by Litwin et al. (2017), which indicates that experiential avoidance mediates the relationship between negative emotions and emotional eating [81]. Similarly, the meta-analysis carried out by Leppanen et al. (2022) demonstrated a strong association between emotional avoidance processes and eating disorders, thus highlighting the importance of a transdiagnostic approach to eating disorders [82]. Another study conducted by Fahrenkamp et al. (2019), aiming to analyze whether experiential avoidance mediated the relationships between food cravings, impulsive eating, and eating-related problems, revealed that experiential avoidance is a factor that influences the relationships between emotional eating, food cravings, and binge eating, which are often associated with unhealthy food choices [83]. In summary, experiential avoidance appears to be an important factor influencing eating behavior. The processes of experiential avoidance can mediate and determine a person’s eating behavior when they are exposed to aversive events (internal or external), which, in the pursuit of immediate well-being, can lead individuals to solidify unhealthy eating patterns and increase the risk of eating disorders and other associated metabolic diseases.

### 5.2. Reward Centre and Metabolic Diseases

While food itself can be a comforting stimulus, the previous paragraphs intentionally refer to hyperpalatable foods, as the literature focuses on them as a central point of metabolic diseases associated with alterations in eating behavior due to their impact in terms of functional and structural changes in individuals. Taste is one of the main determinants of food choice. However, repeated exposure to highly processed and high-fat, high-sugar foods can lead to desensitization of taste receptors, reducing the ability to perceive and enjoy more natural and healthy flavors [84,85,86]. This disruption of taste mechanisms can lead to a preference for hyperpalatable foods, such as snacks, fast food, and ultra-processed products. These hyperpalatable foods are highly appealing and satisfying, as they often contain a combination of fats, sugars, and salt in proportions that stimulate reward centers in the brain.

Numerous studies have shown that foods high in fat, sugar, or salt (highly palatable foods) activate reward responses in the brain, contributing to the capability to activate the brain’s dopaminergic systems, thus creating associations between those foods and pleasurable emotions [87,88,89]. In other words, they become a highly attractive and accessible option for someone seeking immediate relief from an aversive experience. Additionally, there is evidence that excessive nutrition or high caloric intake is associated with structural and functional alterations in the brain [90], such as hypothalamic inflammation and gliosis, reduced brain volume, decreased regional blood flow, or decreased hippocampal size. These induced changes collectively translate into a vicious cycle of disrupted metabolic control and cognitive deficits.

For example, a study conducted by Stice et al. (2008) found that adolescents with greater activation of reward circuits in response to high-fat and high-sugar food images had a higher risk of weight gain in a one-year follow-up. This suggests that the activation of brain reward mechanisms in response to junk food may be a risk factor for the development of obesity and other metabolic diseases [90]. Furthermore, systematic exposure to junk food can have detrimental effects on appetite regulation and metabolism. Studies have shown that frequent consumption of high-fat and high-sugar foods can lead to insulin resistance, increased triglyceride levels, and the accumulation of visceral fat, contributing to the development of metabolic diseases such as type 2 diabetes and cardiovascular disease [91,92].

When attempting to comprehend metabolic disorders in a comprehensive and transdiagnostic manner, it is crucial to analyze the different factors involved in the onset, development, and maintenance of the disease, with particular attention to the experiential avoidance process based on junk food, which can become a vicious cycle leading to metabolic damage. Frequent consumption of junk food, with its high-calorie density and deficiency in essential nutrients, can lead to weight gain, obesity, and the development of metabolic diseases such as type 2 diabetes, cardiovascular disease, and insulin resistance. Furthermore, the combination of food restriction followed by episodes of binge eating junk food, characteristic of disorders such as bulimia nervosa or binge eating disorder, further exacerbates the effects of ineffective management of private events.

## 6. Movement, Physical Activity, and Metabolic Diseases

Hypertension, central obesity, insulin resistance, and atherogenic dyslipidemia are all components of the metabolic illness cluster, which is significantly associated with an elevated risk of developing diabetes and atherosclerotic and nonatherosclerotic (CVD). Both genetic and acquired factors contribute to the ultimate path of inflammation that leads to CVD in the pathogenesis of these disorders. In particular, metabolic syndrome (MetS) has acquired prominence in recent years due to the exponential rise of obesity worldwide. Early diagnosis is essential in order to modify lifestyle and risk factors [11,92]. It has been reported that MetS prevalence is a significant public health concern. MetS is estimated to affect nearly 35% of all adults and 50% of those aged 60 or older in the United States [93] and nearly 31% in Spain [94]. Given that the aging of the world’s population is a significant factor in the increasing prevalence of MetS, this information is alarming. Otherwise, the total cost of the MetS, including the cost of health care and the loss of potential economic activity, is measured in trillions, and costs are expected to rise in the future.

### 6.1. Physical Activity

Traditionally, lifestyle changes (such as healthy eating and regular exercise), pharmaceutical or even surgical approaches (such as bariatric surgery when necessary), and each individual symptom have been recommended for the management of MetS [95]. CVD risk factors can be decreased by improving cardiorespiratory fitness through exercise therapies, and obesity can be decreased with nutritional interventions. In addition, it has been shown that physical activity interventions are efficacious at 12 weeks or more for cardiometabolic parameters [96]. In this regard, exercise has a profound effect on metabolism, and it is crucial that the body adjusts to the new normal in order to produce the ATP that powers muscle contraction and other critical physiologic processes [97]. Under homeostatic regulation, quick and coordinated changes in the secretion of multiple hormones ensure that energy is made available via the appropriate pathways for the intensity and duration of activity. The method of muscle activation is connected to the molecular processes that regulate muscle function and fiber phenotype. In relation to this, there is a continuum between the two primary categories of exercise that we use to categorize physical activity: endurance, commonly also named aerobic exercise, and strength, commonly named resistance [98,99].

#### 6.1.1. Types of Exercise and Metabolic Diseases

**Figure 3:**
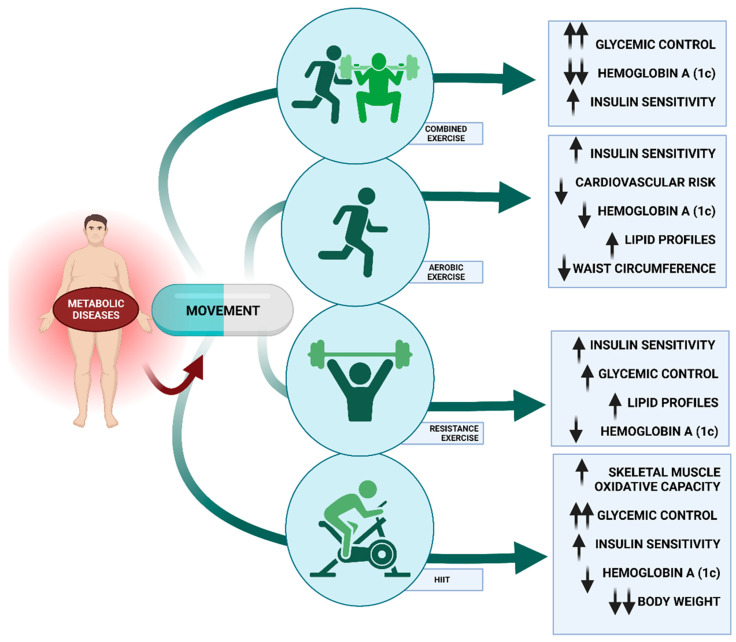
Description of the different improvements that can be achieved through movement and exercise in the different markers of metabolic diseases. Aerobic exercise, strength training, and combined exercise along with HIIT are the most successful approaches. Up arrow-increase; down arrow-decrease.

Chronic adaptations, such as enhanced exercise capacity and altered energy metabolism, may be caused by consistent exercise in addition to the acute alterations induced by a single exercise session [100]. In relation to this, improved VO2max cardiac output from moderate to intense aerobic exercise training (65–90% of maximum heart rate) is linked to significantly lower cardiovascular and total mortality risk in people with TD2 [101]. Moreover, regular aerobic exercise training improves glycemia in these individuals, as evidenced by fewer daily hyperglycemic excursions and 0.5–0.7% reductions in hemoglobin A1C (HbA1c) (Figure 3) [102]. Even without weight loss, regular exercise enhances insulin sensitivity, lipids, blood pressure, other metabolic parameters, and fitness levels. Furthermore, improvements of 10% to 15% have been documented in strength, bone mineral density, blood pressure, lipid profiles, cardiovascular health, insulin sensitivity, and muscle mass in studies examining the impact of resistance training in TD2 (Figure 3) [103]. In addition, resistance training can also enhance older individuals’ health due to the increased prevalence of type 2 diabetes with age and the age-related loss of muscle mass, known as sarcopenia [104]. In summary, improved glucose regulation is made possible through exercise training, whether it be resistance training or aerobic exercise.

As shown above, the evidence indicates that movement is advantageous (Figure 3); however, we need specific information regarding the type of exercise. Some studies, such as Reddy et al.’s, also demonstrate that resistance training is a promising strategy that may result in improved glycemic control, but the results for aerobic training are not as conclusive [105]. However, Sigal et al. showed that either aerobic or resistance training alone enhances glycemic control in type 2 diabetes, but combined aerobic and resistance training provides the greatest improvement. [106] For instance, a randomized controlled trial demonstrated that a combined training protocol implemented with 262 sedentary men and women with type 2 diabetes improved their HbA1c levels after 9 months of training [107]. Similarly, Hai-Guo et al. (2016) specified that although both aerobic exercise and aerobic exercise combined with resistance training for 24 weeks increased the quality of life in type 2 diabetes patients, the combined training was more beneficial than aerobic exercise alone (Figure 3) [108]. In this regard, Schwingshackl and colleagues found that combined training led to a significantly greater reduction in HbA1c than aerobic or resistance training alone after conducting a systematic review of 14 randomized controlled trials for the same three exercise modalities in 915 adults with diabetes [109]. However, in order to compare the metabolic effects of aerobic, resistance, and combination exercise on a total of 1003 people with diabetes, Snowling and Hopkins conducted a head-to-head meta-analysis of 27 controlled trials. They concluded that the variations across exercise modalities were negligible, and all 3 exercise modes had positive effects on HbA1c, fasting and postprandial glucose levels, insulin sensitivity, and fasting insulin levels [110]. However, a more recent review pointed out that weight loss combined with a combination of aerobic and resistance training has been shown to be the most beneficial in improving the functional status of obese older persons [111]. Nevertheless, Earnest et al. showed that not only combination training improved metabolic syndrome scores and prevalence in T2D patients but also aerobic training [112]. Additionally, comparing resistance training and aerobic training samples, Ramalho et al. revealed that there were no alterations in glycated hemoglobin, lipid profile, fasting glucose level, or body mass index (BMI) in aerobic exercises, but there was a reduction in waist circumference and average self-monitored blood glucose levels. In the resistance group, none of the evaluated parameters changed [113].

Overall, the immediate metabolic effects of exercise are mostly independent of insulin, but exercise training can increase insulin sensitivity in the muscles, making it a valuable tool for preventing and treating metabolic diseases [114]. As shown, numerous studies have demonstrated that physical activity is beneficial to health. Using the outpatient clinics at a single academic medical facility, Jarvie et al. recruited 150 patients with medically managed type 2 diabetes and atherosclerotic cardiovascular disease (ACVD) or risk factors for ACVD. Patients with type 2 diabetes who have or are at risk for ACVD often have low blood pressure and low levels of cardiovascular fitness [115]. In this regard, Pan et al. (2018) added that, compared to aerobic or resistance exercise alone, combined exercise resulted in a more significant reduction in HbA1c levels; however, some cardiovascular risk factors showed a less significant reduction. Additionally, there were no significant differences between the combined aerobic and resistance exercises with regard to weight loss [116]. Regarding exercise timing and dietary considerations for weight loss, studies have looked at the best times to exercise before and after meals and generally to improve blood glucose control and other health outcomes in T2D [117]. Although dietary eating habits may be employed to improve blood glucose control, it is still unclear how they would affect exercise. Generally, postprandial exercise improves glucose control by reducing acute glycemic spikes, and greater energy expenditure postprandially reduces glycemia regardless of exercise intensity or type, with a prolonged duration (>45 min) providing the most consistent benefits [118]. Thus, although weight loss from physical activity alone is modest, it is possible with 1 or more hours of moderate- or vigorous-intensity exercise per day [119]. Weight loss in obese men and women who engaged in aerobic exercise for 1 hour per day was comparable to that achieved with dietary restriction alone, and both groups experienced reductions in abdominal subcutaneous and visceral fat [120,121]. Consistent exercise inhibits weight growth and decreases both subcutaneous and visceral fat. Moderate weight loss with dietary restriction alone or diet plus exercise similarly reduced total abdominal fat, subcutaneous adipose tissue, and glycemia in postmenopausal women with T2D, but the addition of exercise was required for visceral adipose tissue loss, resulting in reduced metabolic dysfunction and CVD risk [118]. Thus, moderate-to-vigorous exercise (500 kcal) performed 4–5 times per week appears to reduce visceral fat in adults with T2D and may reduce their metabolic risk [118].

#### 6.1.2. Intensity and Metabolic Diseases

Regarding exercise’s intensity, it has been demonstrated that both high-intensity interval training (HIIT) and moderate-intensity continuous training (MICT) are feasible, well-tolerated, and safe, and they produce comparable improvements in aerobic fitness in middle-aged and elderly individuals with type 2 diabetes [122]. These findings have significant implications for the prescribing of exercise to diabetic patients. In recent years, high-intensity interval training (HIIT) has become one of the workout programs with the quickest growth rates [123]. HIIT consists of four to six repetitions of 30-s bursts of maximum intensity separated by 30- to 60-s rest or active recovery periods. A single session of exercise usually lasts for 10 min and is performed on a stationary bike (Figure 3). In people with type 2 diabetes, HIIT improves skeletal muscle oxidative capacity, glycemic management, and insulin sensitivity [123,124]. A recent meta-analysis evaluated the effects of HIIT programs on glucose management and insulin resistance and concluded that HIIT had superior results compared to aerobic training or doing no exercise as a control. In 50 trials with interventions lasting at least 2 weeks, people in the HIIT group lost an average of 1.3 kilograms and 0.19% of their body weight compared to those in the control group [123,124]. However, Fisher et al. specified that when comparing moderate intensity continuous training (MICT) versus HIIT, although the majority of the cardiometabolic risk factors evaluated showed similar increases with both, MICT showed a larger improvement in overall cardiovascular fitness. Nevertheless, these findings indicate that overweight or obese young men who were previously sedentary may benefit from a very brief period of either HIIT or MICT training [125]. In this regard, a previously referenced meta-analysis found that 34% of the studies based on HIIT protocols documented adverse events, the bulk of which were attributed to musculoskeletal injuries sustained during HIIT rather than moderate training [123].

However, numerous studies have demonstrated that a variety of obstacles invariably result in the discontinuation of exercise training programs and regular physical activity, leading to the development of MetS and, ultimately, T2D and cardiovascular disease (CVD) [94]. The translation of efficacy studies demonstrating the benefits of a wide range of exercise and activity modalities is essential. Studies suggest that supervised training programs are more effective than unsupervised training programs. On the other hand, it is hypothesized that since exercise (and other lifestyle therapies, such as diet) are difficult to maintain for many, it will be simpler for individuals to maintain health improvements through drug therapy [126]. Exercise has the potential to lessen the health burden of diabetes complications such as nephropathy, retinopathy, neuropathy, and peripheral arterial disease, and future clinical research will increase our understanding of the interactions (positive and negative) between exercise and diabetes medications [127].

## 7. Nutrition and Metabolic Diseases

One of the ultimate objectives of the promising field of precision nutrition is the development of individualized dietary recommendations for the treatment or prevention of metabolic disorders [128]. Specifically, precision nutrition seeks to develop more comprehensive and dynamic nutritional recommendations based on changing and interacting parameters in an individual’s internal and external environment over the course of their lifetime. In addition to genetics, precision nutrition approaches take into account dietary preferences, food behavior, physical activity, the microbiome, and the metabolome [129,130]. However, emerging translational evidence suggests that epigenetic alterations (DNA methylation, miRNA expression, and histone modifications) occur in response to external stimuli and may contribute to heightened inflammation and the risk of developing a variety of diseases, such as diabetes, cardiovascular disease, cancer, and neurological disorders [131]. Thus, there is a correlation between nutritional factors and pro-inflammatory potential [132].

### 7.1. High-Fat Diets and Metabolic Diseases

**Figure 4:**
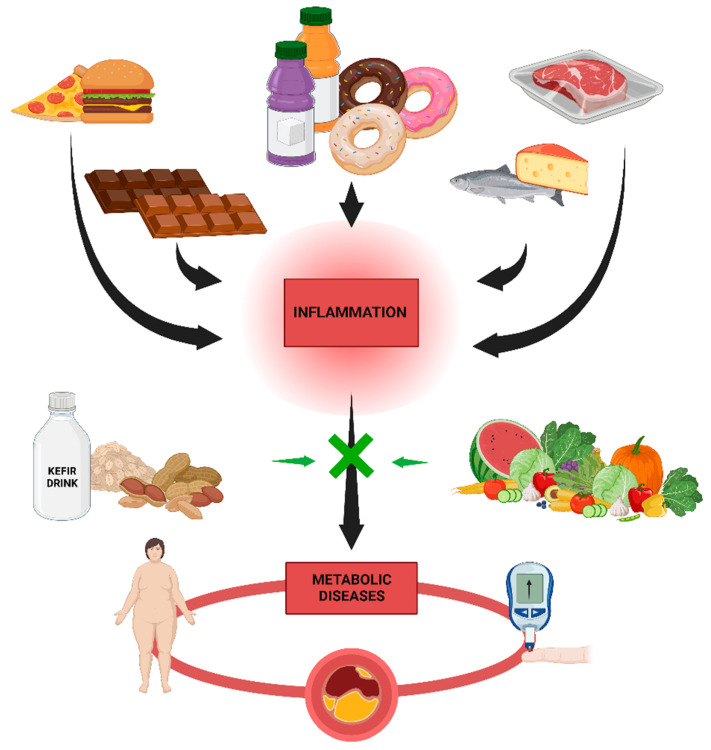
Description of the types of diet that can cause inflammatory processes including animal fats, chocolates, ultra-processed and sugary products. Vegetables, fruits, nuts, pre- and probiotics and cereals are shown as foods that can curb or prevent inflammation and thus the development of metabolic diseases.

Specifically, the consumption of Western-style diets induces a state of chronic metabolic inflammation, or "metainflammation," which contributes to the onset of numerous prevalent noncommunicable diseases (NCDs) [133]. Christ et al. showed in a comprehensive review that the development of effective preventive and therapeutic methods for prevalent NCDs requires a deeper understanding of how modern lifestyles and the Western diet activate immune cells [133]. Particularly in Western countries, metabolic illnesses such as obesity, type 2 diabetes, and others have a major impact on world health. Chronic low-grade inflammation is largely caused by dysmetabolism, which is characterized as the failure to maintain homeostasis and leads to loss of lipid regulation, oxidative stress, inflammation, and insulin resistance (Figure 4) [134,135,136]. In relation to this, chronic low-grade inflammation can be present in one or multiple organs, despite not yet being detectable in the bloodstream [137]. The response to a metabolic challenge containing lipids may amplify dysfunctionalities at the tissue level, resulting in an excess of inflammatory markers in the circulation, thereby facilitating the detection of early low-grade inflammation [138].

Concretely, dietary macronutrients can induce inflammation by activating the Toll-like receptor 4 (TLR4) of the innate immune system. For instance, long-term consumption of a high-fat diet (a diet in which at least 35% of total calories come from fats, both saturated and unsaturated), contains at least 35% lipids. In addition to processed foods, many other foods, including but not limited to animal fat, chocolate, butter, and oily seafood, have a high-fat content [139]. High-sugar and high-meat diets such as the Western Diet appear to induce chronic low-grade systemic inflammation, endotoxicity, and metabolic diseases (Figure 4) [140,141]. In this regard, people who consume large quantities of ultra-processed foods are more likely to be obese than those who consume comparatively small amounts, and the availability of ultra-processed foods is positively correlated with the prevalence of obesity [142]. However, compared to refined cereals, consumption of whole grains is associated with a reduced risk of several non-communicable diseases [143]. A study on fish consumption found that processed fish has adverse effects on metabolic syndrome markers, while whole fish appears to be protective [144].

### 7.2. Low-Fat Diets

Due to this, the Mediterranean Diet (MD) is a potential treatment for metabolic syndrome because it prevents adiposopathy, or “sick fat,” formation [145]. For instance, De Lorenzo et al. examined the effects of MD on the body composition and metabolic profile of 19 obese women. Total fat mass and segmental fat mass from the trunk and legs decreased substantially following a 2-month MD regimen, whereas neither total nor segmental lean body mass decreased significantly [146]. At least in part, the apparent ability of traditional medicine to reduce the risk of developing and progressing cardiovascular disease, cancer, and degenerative diseases has been attributed to the nutraceutical effect of micronutrients and compounds with antithrombotic, anticancer, and antioxidant properties [147]. Carbohydrates are dominated by starch, which is derived primarily from wheat (bread, pasta) and to a lesser extent from other cereals and pulses, while the proportion of sucrose for moderate consumption of sugar and desserts is extremely low [145]. In relation to this, greater adherence to the MD is associated with higher antioxidant levels and lower oxidized LDL-cholesterol concentrations, suggesting a beneficial effect of the MD on cardiovascular health (Figure 2) [147]. In contrast, chronic consumption of a Western diet coupled with sedentary behavior results in chronic metabolic inflammation (termed metaflammation), which is “remembered” by innate immune cells via long-lasting metabolic and epigenetic cellular reprogramming [148]. However, a review conducted by Kratz et al. (2013) reported that intake of high-fat dairy products was inversely associated with measures of body fatness. According to studies examining the relationship between high-fat dairy consumption and metabolic health, either an inverse association or no association was found [149]. Conversely, Giugliano and colleagues demonstrated that the most important dietary strategies to reduce, for instance, cardiovascular diseases include adequate ingestion of omega-3 fatty acids, reduction of saturated and trans-fats, and consumption of a diet rich in fruits, vegetables, nuts, and whole grains but deficient in refined grains (Figure 2). Each of these strategies may be associated with reduced inflammation production [150]. However, high-fat diets, such as the Western Diet, are associated with all-cause mortality, cardiovascular disease, coronary heart disease, ischemic stroke, or type 2 diabetes [151]. Numerous prospective studies have discovered associations between fat consumption and the risk of developing T2D [152]. In a four-year prospective diabetes study, more than a thousand subjects without a previous diabetes diagnosis were evaluated. In this investigation, researchers discovered a link between fat consumption, T2D, and impaired glucose tolerance [153].

As mentioned in previous subsections, MetS is a cluster of metabolic abnormalities that raises the risk of atherosclerotic cardiovascular disease and type 2 diabetes. It is a complex interaction of genetic, metabolic, and environmental factors, although the precise cause is unknown. Dietary practices are the most important environmental factor in the prevention and treatment of this condition [153]. The evidence suggests that the components of the “healthy” diet that are currently recommended are likely also protective against MetS, including low saturated and trans fat (rather than low total fat) and balanced carbohydrate intake rich in dietary fiber, as well as high fruit and vegetable intake (rather than low total carbohydrate) and the inclusion of low-fat dairy foods (Figure 2) [154]. For instance, Marrone et al. specified that a vegan diet reduces the risk of chronic non-communicable degenerative diseases such as MetS [155]. Additionally, MetS patients may benefit from plant-based diets that are nutritionally sound. The different types of plant-based diets (vegan, lacto-vegetarian [156], lacto-ovo-vegetarian [157], and pescatarian [158]) are discussed with an emphasis on the specific effects of dietary components on weight maintenance, protection against dyslipidemias, insulin resistance, hypertension, and low-grade inflammation [159].

Recent research supports the concept that intestinal microorganisms play a role in the host’s metabolism as well as the preventative and therapeutic potentials of probiotic and prebiotic interventions for metabolic diseases. Specific intestinal bacteria appear to function as lipopolysaccharide (LPS) sources via LPS and/or bacterial translocation into the circulation as a result of a compromised microbial barrier and increased intestinal permeability and to play a role in systemic inflammation and the progression of metabolic diseases [140]. Thus, probiotic or fermented foods may contain beneficial bacterial molecules left over from fermentation [159]. Studies have shown that fermented milk products have beneficial effects on metabolic markers in mice [160], regardless of the presence of live probiotic bacteria in the product or the recipient’s intestines. In vitro studies have shown that metabolites from probiotic bacteria can inhibit the production of pro-inflammatory cytokines (Figure 2) [161]. Particularly, pre- and probiotics have emerged as efficient and integrative means of modulating the microbiome to rectify the microbial dysbiosis associated with an obese phenotype [162].

## 8. Single-Cell Transcriptomics in Metabolic Diseases

Metabolic diseases are complex disorders characterized by altered metabolic pathways and cellular processes that contribute to tissue dysfunction and organ damage. The molecular mechanisms underlying metabolic diseases are not fully understood, and current therapies are often inadequate. Single-cell transcriptomics is a powerful tool for investigating the molecular basis of disease as it allows the examination of gene expression patterns in individual cells, providing insights into cellular heterogeneity and the molecular mechanisms underlying metabolic diseases [163]. Single-cell transcriptomics is an emerging field of molecular biology that has the potential to provide unprecedented insight into metabolic diseases. Metabolic diseases are caused by mutations in metabolic pathways, leading to the inability of cells to efficiently process energy and nutrients. Single-cell transcriptomics enables the study of individual cells by providing a snapshot of gene expression at the single-cell level. This technique is particularly useful for studying metabolic diseases, as it can provide insight into the expression patterns of metabolic genes and their regulation in response to disease.

Single-cell transcriptomics has been used to study several metabolic diseases, such as diabetes, obesity, and fatty liver disease. In these studies, single-cell transcriptomics allowed investigators to identify cellular subtypes, which allowed a better understanding of the disease pathogenesis. Additionally, single-cell transcriptomics has allowed researchers to identify novel therapeutic targets, which may lead to the development of new treatments for these diseases [164]. The use of single-cell transcriptomics in metabolic diseases has several advantages over traditional methods of studying metabolic diseases. For example, single-cell transcriptomics enables the study of the metabolic pathways of individual cells, which is not possible with bulk sequencing techniques [165]. Additionally, single-cell transcriptomics can provide a more detailed picture of the expression of metabolic genes in response to disease [166]. Finally, single-cell transcriptomics is a relatively low-cost method that can be used to identify potential therapeutic targets for metabolic diseases in a cost-effective manner [167].

Single-cell transcriptomics has been used to identify previously unknown cell subtypes in adipose tissue [168] and to investigate the heterogeneity of liver cells in non-alcoholic fatty liver disease [169]. In pancreatic islets, single-cell transcriptomics has revealed subpopulations of beta cells with distinct functional properties [170]. These findings demonstrate the power of single-cell transcriptomics in understanding the complexity of cell types and states in metabolic diseases. Another study used single-cell transcriptomics to investigate the heterogeneity of adipose tissue in obese individuals with and without type 2 diabetes (T2D) [171]. The study identified certain cell types that were associated with T2D status, suggesting that targeting specific cell types may be a promising therapeutic strategy for T2D. Single-cell transcriptomics has also been used to investigate the immune response in metabolic diseases. For instance, a study used single-cell transcriptomics to examine the immune cells present in adipose tissue in obesity [172]. The study identified distinct subpopulations of immune cells, including T cells and macrophages, that displayed unique transcriptional signatures associated with obesity and insulin resistance. Another study used single-cell transcriptomics to investigate the immune cells present in the islets of Langerhans in type 1 diabetes (T1D) [17]. The study revealed the presence of previously unknown immune cell subtypes and identified transcriptional changes associated with T1D progression. Single-cell transcriptomics has also been applied to study metabolic diseases in model organisms. A recent study used single-cell transcriptomics to examine the liver cells of mice fed a high-fat diet [173]. The study revealed transcriptional changes in specific cell types, including hepatocytes and immune cells, which may contribute to the development of non-alcoholic fatty liver disease.

At the cellular level, metabolic diseases can be caused by a variety of factors, such as genetic mutations, epigenetic modifications, and the environment [174]. Single-cell transcriptomics can provide insights into the molecular changes occurring in individual cells, which can in turn inform our understanding of the underlying mechanisms of metabolic diseases. For example, single-cell transcriptomics can be used to identify subpopulations of cells that are differentially expressed in metabolic diseases, as well as to identify gene pathways that are perturbed in disease states [17]. Furthermore, single-cell transcriptomics can be used to study how metabolic diseases progress over time, as well as how they interact with other diseases. This could provide insight into how metabolic diseases are affected by lifestyle and environmental factors, as well as how they interact with other diseases [175].

Single-cell transcriptomics has been used to study a variety of metabolic diseases, including diabetes, obesity, and fatty liver disease. Single-cell transcriptomics can reveal changes in gene expression that are associated with metabolic diseases, such as changes in the expression of genes involved in insulin signaling, glucose metabolism, and lipid metabolism [176]. By understanding the changes in gene expression associated with metabolic diseases, researchers can develop targeted treatments that modulate gene expression and improve patient outcomes [177]. In addition, single-cell transcriptomics can be used to study the effects of environmental factors on gene expression in cells associated with metabolic diseases. For example, researchers have used single-cell transcriptomics to study the effects of diet and exercise on gene expression in cells associated with type 2 diabetes [178].

Recent studies using single-cell transcriptomics have provided new insights into the molecular mechanisms underlying metabolic diseases. For example, a study by Wang et al. (2020) used single-cell RNA sequencing to examine the gene expression patterns in pancreatic islets from patients with type 2 diabetes. The study identified novel cell types and transcriptional programs associated with disease progression, providing potential targets for therapeutic intervention. Another study by Jin et al. (2021) used single-cell RNA sequencing to investigate the effects of high-fat diet-induced obesity on adipose tissue. The study found that obesity is associated with the upregulation of genes involved in inflammation and immune response, providing a link between obesity and chronic inflammation. In addition, single-cell transcriptomics can be used to study the effects of environmental factors on gene expression in cells associated with metabolic diseases, allowing for the development of personalized treatments and interventions tailored to the individual patient [179].

Single-cell transcriptomics is a powerful tool for studying metabolic diseases. This technology has allowed researchers to gain a better understanding of the pathogenesis of metabolic diseases as well as identify novel therapeutic targets. The use of single-cell transcriptomics in metabolic diseases is expected to continue to progress and may lead to the development of new treatments for these diseases.

## 9. Gut Microbiota in Metabolic Diseases

The gut microbiota refers to a community of microorganisms that inhabit the digestive system of mammals, encompassing various sections from the stomach to the colon. This intricate and ever-changing network consists of approximately 500 to 1000 distinct species, totaling around 1014 cells. Remarkably, this microbial ecosystem surpasses the number of cells in the human body by a factor of ten. It constitutes a symbiotic superorganism, comprising both eukaryotic and prokaryotic cells that collaborate to uphold the overall well-being of the host organism. Notably, the composition of the gut microbiota is highly personalized, differs from one individual to another, and undergoes fluctuations over time [180]. Several factors, including genetics, diet, age, medication, and exposure to the environment, exert influence on its makeup and functioning [181]. Recent studies link and attribute a powerful influence on the development of chronic diseases, including metabolic syndrome, to the microbiota [182]. However, understanding the precise role of the microbiome in metabolic diseases has remained a challenge.

In this line, recent research has started to elucidate the specific mechanisms through which the gut microbiome may influence metabolic health. For instance, studies have highlighted the role of gut microbiota-derived metabolites, such as short-chain fatty acids, in modulating host metabolism and energy homeostasis [183]. Additionally, investigations have uncovered links between gut dysbiosis and metabolic disorders, implicating imbalances in microbial diversity and composition in the development of conditions such as obesity, insulin resistance, and dyslipidemia [184]. Understanding these intricate connections could pave the way for novel therapeutic approaches targeting the gut microbiome to prevent or manage metabolic diseases.

### 9.1. Gut Microbiome and Obesity

In this line, the prevalence of obesity has been steadily increasing worldwide, leading to the recognition of metabolic syndrome as a significant health issue. Only in the United States has the percentage of overweight adults risen from 15% before 1980 to a documented high of 39.8% in 2015–2016, indicating a substantial 25% increase over 35 years [185]. This alarming rise in obesity rates, along with the link between central obesity, type 2 diabetes, and cardiovascular disease, has raised concerns about the “epidemic” nature of the problem. While obesity was previously attributed to an imbalance between energy intake and expenditure, recent research reveals that weight regulation is a complex process influenced by various factors such as genetics, lifestyle choices, metabolic processing of foods, and the gut microbiome [186].

The authors demonstrated the impact of diet on the modulation of the human gut microbiome, suggesting alterations in gut microbial communities based on whether individuals follow a plant-based or animal-based diet. Animal-based diets tend to favor the growth of bile-tolerant microbes while reducing the abundance of fiber-fermenting bacteria [187]. Additionally, the composition of dietary fats, types of fiber, and food additives also impact the gut microbiome [188]. Certain dietary fats possess antimicrobial properties, and different diets can lead to the proliferation of specific microbial species, some of which have been associated with obesity-related conditions. For example, the presence of the proinflammatory bacteria Bilophila wadsworthia has been linked to a diet rich in lard [189], while Lactobacillus and Akkermansia muciniphila have been associated with a fish oil-based diet [190]. Interestingly, A. muciniphila has shown a negative correlation with obesity, type 2 diabetes, and hypertension. In a human trial, oral supplementation of A. muciniphila improved insulin sensitivity and reduced insulinemia and plasma total cholesterol in overweight or obese individuals with insulin resistance [191]. These findings highlight the role of diet and the gut microbiome in both the development of obesity and potential therapeutic approaches. Furthermore, rodents fed diets high in fermentable dietary fiber were found to be protected against diet-induced obesity and metabolic disorders. The authors suggest that the fermentation of fiber by gut microbes produces metabolites that stimulate the production of beneficial peptides such as glucagon-like peptide-1 (GLP-1) and GLP-2, which have positive effects on glucose metabolism and intestinal health [192].

In addition to diet, external cues from the environment can impact the composition and behavior of the gut microbiome, exacerbating the detrimental effects of high-fat, high-sugar Western diets [193]. In this line, manipulation of the light/dark cycle through factors such as overnight shift work and international travel interferes with the body’s natural circadian rhythms, which evolved based on the 24-h rotation of the Earth [194]. Many biological processes, including hormone release, blood glucose levels, and gut function, follow cyclical patterns during a 24-h period. Disruptions to this natural cycle have been associated with obesity, impaired insulin sensitivity, and altered lipid metabolism [195]. In animal models, Leone et al. found that germ-free mice, regardless of whether they were fed a low-fat or high-fat diet, lacked the typical diurnal expression of clock genes in the brain and liver. In contrast, conventionally raised mice with normal gut microbiota maintained the regular diurnal expression of clock genes, but only when they were fed a low-fat diet [196]. When these mice were fed a high-fat diet, circadian disruption occurred. Additionally, Thaiss et al. demonstrated in mice that the thickness of the mucus barrier in the gut also fluctuates in a cyclical manner, corresponding to changes in microbial abundance [197]. These findings suggest that disturbances in the circadian rhythms of the gut microbiome could potentially lead to defects in the gut barrier.

### 9.2. Gut Microbiome and Dyslipidemia

Dyslipidemia refers to an abnormal lipid profile in the blood and is a significant risk factor for cardiovascular disease. It is considered a diagnostic criterion for metabolic syndrome. Its implications for chronic diseases are related to impaired glucose metabolism, obesity, and atherosclerotic plaques, thus posing a significant risk for cardiovascular disease [198]. Its relationship with the gut microbiota is in terms of the short-chain fatty acids (SCFAs) produced by microbial fermentation of dietary fibers, which have shown potential in mitigating the effects of dyslipidemia [199].

In this line, recent reviews strongly suggest that microbial metabolites, specifically short-chain fatty acids produced through the fermentation of dietary fiber, have the potential to protect against the development of dyslipidemia and metabolic syndrome [200]. Butyrate, for instance, supports the health and integrity of the intestinal epithelial cells and has been found to be lower in individuals with inflammatory diseases [201]. Moreover, butyrate supplementation has shown positive effects on intestinal gluconeogenesis, food intake, and glucose metabolism [202]. While SCFAs demonstrate protective effects, other bacterial metabolites can have different implications. Trimethylamine (TMA), secondary bile acids, and components of the bacterial cell wall such as lipopolysaccharide (LPS) are examples of such metabolites. These metabolites may act as drivers or strong contributors to dyslipidemia and metabolic syndrome [203]. For instance, studies have shown that TMA, produced by gut bacteria from dietary phosphatidylcholine, can be converted into trimethylamine N-oxide (TMAO), a pro-atherosclerotic molecule associated with atherosclerosis. Furthermore, certain components of the bacterial cell wall, such as LPS, have been linked to cardiovascular disease risk as they can trigger immune responses and contribute to metabolic disorders [204].

Taken together, the findings suggest that the balance between beneficial SCFAs and potentially harmful bacterial metabolites, such as TMAO and LPS, may play a crucial role in dyslipidemia and metabolic syndrome. Yet, further research is needed to fully understand the intricate interactions between the gut microbiome, microbial metabolites, and metabolic disorders.

### 9.3. Gut, Inflammation and Insulin Resistance

Chronic low-grade inflammation is not officially recognized as a defining factor of metabolic syndrome, but it does play a significant role in the development of obesity and insulin resistance, which are closely associated with metabolic syndrome. The intestinal permeability and the microbiome have a central role in chronic low-grade inflammation, making them crucial contributors to the metabolic abnormalities observed in the metabolic syndrome [186].

The authors reported an elevation of proinflammatory markers in the adipose tissue of obese individuals [205]. In mice and humans, enlarged adipocytes showed macrophage infiltration and the presence of crown-like structures consisting of phagocytosing macrophages around dying adipocytes [206]. Both adipocytes and macrophages produce proinflammatory cytokines and chemokines. Furthermore, the authors discovered that a high-fat diet-induced chronic activation of NF-B in the liver, resulting in subacute inflammation, hyperglycemia, and severe hepatic insulin resistance [207]. These studies laid the foundations, showing the interrelationship between established chronic low-grade inflammation as a significant factor in the development of diabetes.

Furthermore, the presence of intestinal pathobionts, which are commensal bacteria that can become pathogenic [208], In this line, authors have pointed out that patients with chronic kidney disease exhibit increased oxidative stress and inflammation by releasing uremic toxins due to an impaired microbiome [209]. Another study intentionally disrupted the natural anaerobic homeostasis of the intestine and found that an oxygenated gut promoted the expansion of pathobionts and induced inflammation [186]. The intestine’s PPAR limits oxygen availability through oxidation in colonocytes, and this process is stimulated by bacterially produced butyrate. When butyrate-producing bacteria were eliminated from the gut, oxygen levels increased, leading to the proliferation of pathogenic forms of E. coli and Salmonella [210].

Additionally, the endocannabinoid system, which is associated with obesity and low-grade inflammation, may be influenced by the gut microbiome [211]. Bacterial lipopolysaccharide stimulates endocannabinoid synthesis, and inhibiting the cannabinoid receptor CB1 has shown protective effects against obesity, hepatic steatosis, and low-grade inflammation [212]. Studies comparing germ-free mice with conventional mice and investigating dietary factors and genetically modified mice with altered gut microbiome composition have demonstrated the role of the microbiome in endocannabinoid activity within the colon and adipose tissue [213].

Regarding altered glycemia, and intestinal permeability, Thaiss et al., revealed that intracellular hyperglycemia in the intestinal epithelium is a key driver of dysmetabolism, among other factors, proposing that dysmetabolism can both contribute to and be a consequence of impaired barrier function [213]. Collectively, these findings suggest that the evolving definition of metabolic syndrome should consider the interplay between chronic hyperglycemia, intestinal permeability, and the heightened risk of systemic infections.

### 9.4. Future Lines of Intervention

Fecal microbiota transplants (FMT) are one of the direct approaches recently being used and investigated that show a direct impact of the microbiome on traits. This procedure involves transferring fecal matter from a donor to a recipient using different methods. FMT has demonstrated significant efficacy in treating colitis caused by recurrent Clostridium difficile infections. Although the precise mechanism is not fully understood, FMT restores the recipient’s microbiome diversity, which gets depleted due to the long-term use of broad-spectrum antibiotics [214]. By displacing C. difficile in the intestine, FMT effectively eliminates the infection. This success prompts the question of whether FMT can be utilized for other purposes, such as transferring a metabolically healthy microbiome to individuals with metabolic syndrome in order to confer a healthy phenotype [215].

Animal studies have shown promise in this regard, illustrating that transferring microbiota from normal mice to germ-free mice results in increased body fat content and insulin resistance [216]. In humans, the transfer of microbiota from lean donors to obese individuals with metabolic syndrome has led to improved insulin sensitivity and increased levels of beneficial bacteria within a short period of time [217]. However, longer-term observations have revealed that the gut microbiota composition and insulin sensitivity eventually return to baseline levels [218]. These findings suggest the existence of a metabolically healthy microbiome and indicate the potential for transferring the associated phenotype to individuals with metabolic syndrome through FMT. However, further studies with larger sample sizes and longer durations are required to assess the long-term stability of donor engraftment and the associated phenotypes.

## 10. Epigenetics and Metabolic Diseases

Epigenetics refers to changes in gene expression without changes in the underlying DNA sequence. These changes can be stable over time and passed on to future generations. Metabolic diseases, such as obesity and type 2 diabetes, have a complex etiology that involves both genetic and environmental factors [219]. Recent research has shown that epigenetic modifications play an important role in the development of metabolic diseases. In this discussion, we will explore the relationship between epigenetics and metabolic diseases and discuss some of the mechanisms involved. Epigenetic modifications can occur through several mechanisms, including DNA methylation, histone modifications, and non-coding RNA molecules [220]. DNA methylation is the addition of a methyl group to a cytosine residue in the DNA sequence, which can affect gene expression by inhibiting transcription. Histone modifications, such as acetylation and methylation, can also affect gene expression by changing the accessibility of DNA to transcription factors. Non-coding RNA molecules, such as microRNAs, can bind to messenger RNA and inhibit translation [221]. Studies have shown that epigenetic modifications can be influenced by environmental factors, such as diet and exposure to toxins [222]. For example, maternal diet during pregnancy has been shown to affect DNA methylation patterns in the offspring, which can increase the risk of metabolic diseases later in life [223]. Similarly, exposure to endocrine-disrupting chemicals, such as bisphenol A (BPA), can alter DNA methylation patterns and increase the risk of obesity and diabetes [224].

Several studies have also investigated the role of epigenetic modifications in the development of metabolic diseases. For example, it was found that DNA methylation patterns in adipose tissue were associated with insulin resistance and obesity [225]. Another study by Rönn et al. (2013) found that DNA methylation patterns in blood were associated with BMI and the risk of type 2 diabetes [226]. In this line, several mechanisms have been proposed to explain the relationship between epigenetic modifications and metabolic diseases. One mechanism is the effect of epigenetic modifications on adipocyte differentiation and function. Adipocytes, or fat cells, play an important role in energy metabolism by storing and releasing fatty acids. Studies have shown that DNA methylation patterns in adipose tissue can affect adipocyte differentiation and function [227]. For example, DNA methylation of the peroxisome proliferator-activated receptor gamma (PPARG) gene, which regulates adipocyte differentiation, has been shown to be associated with obesity and insulin resistance [225].

### 10.1. Epigenetic Modifications on Inflammation

Another mechanism is the effect of epigenetic modifications on inflammation and oxidative stress. Inflammation is a complex process that involves the activation of immune cells and the production of cytokines and other inflammatory mediators [228]. Epigenetic modifications, including DNA methylation, histone modifications, and non-coding RNA expression, can regulate the expression of genes involved in inflammation. For example, DNA methylation of the CXCR4 gene was associated with inflammation in chronic obstructive pulmonary disease (COPD) patients [229]. Also, the histone acetylation of the IL-6 gene was involved in the regulation of inflammation in osteoarthritis [230]. Oxidative stress is a process that occurs when there is an imbalance between the production of reactive oxygen species (ROS) and antioxidant defenses [231]. Epigenetic modifications can affect the expression of genes involved in oxidative stress, including antioxidant enzymes and genes involved in ROS production. For example, previous research found that DNA methylation of the Nrf2 gene was involved in the regulation of oxidative stress in non-alcoholic fatty liver disease [232]. As well, histone methylation of the SOD2 gene was involved in the regulation of oxidative stress in diabetic nephropathy [233]. Inflammation and oxidative stress play a key role in the development of metabolic diseases by promoting insulin resistance and impairing glucose metabolism. Epigenetic modifications can affect the expression of genes involved in inflammation and oxidative stress, such as nuclear factor-kappaB (NF-kB) and superoxide dismutase (SOD) [234]. For example, DNA methylation of the SOD2 gene, which encodes an antioxidant enzyme, has been shown to be associated with insulin resistance and type 2 diabetes [235].

### 10.2. Epigenetic Modifications and Gut Microbiome

Epigenetic modifications can also affect the gut microbiome, which plays a critical role in energy metabolism and glucose homeostasis. Studies have shown that epigenetic modifications can affect the composition and function of the gut microbiome, which in turn can affect the development of metabolic diseases [236]. For example, DNA methylation of the FUT2 gene, which encodes a protein involved in the production of fucosylated glycans in the gut, has been shown to be associated with changes in the gut microbiome and an increased risk of metabolic syndrome [237]. Another study found that maternal high-fat diet-induced changes in DNA methylation patterns in the offspring’s gut epithelium were associated with altered gut microbiota composition and increased susceptibility to obesity and metabolic diseases [238]. Another study found that alterations in histone acetylation patterns in the liver of obese mice were associated with changes in gut microbiota composition and function [239]. Furthermore, epigenetic modifications can affect the gut microbiome by modulating host immune function. For example, a study found that DNA methylation patterns can regulate the expression of AMPs in the gut epithelium. Alterations in DNA methylation patterns can lead to decreased AMP expression, making the host more susceptible to gut pathogens [240]. Epigenetic modifications can also affect the gut microbiome by modulating host signaling pathways. For example, a study found that histone deacetylase inhibitors can alter gut microbiota composition and function in mice [239]. These modifications can alter histone acetylation patterns and gene expression profiles in the host’s cells, leading to changes in metabolism and immune function.

### 10.3. Epigenetic Modifications and Expression of Genes

The epigenetic modifications can also affect the expression of genes involved in energy metabolism and glucose homeostasis, such as insulin signaling and glucose transporters. For example, DNA methylation of the insulin receptor (INSR) gene has been shown to be associated with insulin resistance and type 2 diabetes [241]. Similarly, DNA methylation of the glucose transporter type 4 (GLUT4) gene, which regulates glucose uptake in adipose tissue and muscle, has been shown to be associated with insulin resistance and type 2 diabetes [242]. Histone modifications can also alter the accessibility of genes involved in host-microbe interactions, leading to changes in the gut microbiome. Non-coding RNAs, such as microRNAs and long non-coding RNAs, can also regulate gene expression in the gut microbiome by modulating host signaling pathways [243].

The relationship between epigenetics and metabolic diseases has important implications for the prevention and treatment of these conditions. One potential avenue for prevention is through lifestyle interventions, such as diet and exercise. Several studies have shown that changes in diet and exercise can affect epigenetic modifications and reduce the risk of metabolic diseases [244]. In this line of research, it was found that a Mediterranean diet supplemented with extra virgin olive oil or nuts was associated with changes in DNA methylation patterns and a reduced risk of cardiovascular disease [245]. Another potential avenue for prevention and treatment is using epigenetic drugs, which can modify epigenetic marks and alter gene expression. Several epigenetic drugs are currently in development for the treatment of metabolic diseases, including inhibitors of DNA methyltransferases and histone deacetylases [246]. However, these drugs are still in the early stages of development, and their long-term safety and efficacy have yet to be established.

Finally, it was shown how epigenetic modifications play an important role in the development of metabolic diseases such as obesity and type 2 diabetes. These modifications can be influenced by environmental factors, such as diet and exposure to toxins, and can affect adipocyte differentiation and function, inflammation and oxidative stress, the gut microbiome, energy metabolism, and glucose homeostasis. Lifestyle interventions, such as diet and exercise, and the development of epigenetic drugs may offer new avenues for the prevention and treatment of metabolic diseases.

## 11. Advanced Imaging Techniques and Metabolic Diseases

In the last few years, advanced imaging techniques, including positron emission tomography (PET) and magnetic resonance imaging (MRI), have been proposed as useful tools in order to improve comprehension of endocrine organ pathophysiology as well as metabolic pathways that involve both glucose and lipid metabolism regulation.

Positron emission tomography (PET) has been highlighted by its capability to detect slightly molar amounts of radiotracers, offering consistent quantification of total radiotracer uptake [247,248,249,250]. Thus, the procedure is quite simple. PET is a technique that involves the detection of two coincident gamma rays produced from the destruction of a positron, a particle that has equal electric mass to the electron as well as equal electric charge, but is positive, with an adjacent electron [251,252]. This detection is possible due to the use of a specific tracer, depending on the nature of the analyzed substance, which may be targeted to specific tissues [253,254]. In this line, PET has been underlined as an advantageous technique to study beta cells’ pancreatic functioning. Beta cell dysfunction as well as beta cell mass (BCM) have been proposed as key pathophysiological factors in both diabetes mellitus type 1 (T1DM) and type 2 (T2DM) development, as previous authors suggested [255,256], revealing their increased interest in diabetes prognosis. Consequently, it has been proposed that the study of the viability and mass of BMC could be considered useful in order to highlight diabetes mellitus development, as significant alterations obtained through PET use were found in BCM in both T1DM and T2DM patients. Additionally, PET has also been suggested as a helpful technique that allows visualization of the transplanted cells in pancreatic islet transplantation, an innovative procedure that has shown remarkable outcomes in treating T1DM [253,257]. In this line, previous authors proposed how a connection may exist between these two different types of diabetes, being both linked to destruction in functional BCM, as it has been pointed out how significantly decreased BCM levels were found in T2DM patients [258,259]. Thus, it may be explained due to the fact that hyperglycemia may enhance oxidative stress, leading to apoptosis enhancement, as well as changes in the -cell phenotype, compromising -cell differentiation [260,261]. As a consequence, diabetes progression may be promoted. Moreover, recent literature showed interesting outcomes concerning PET utilization, as PET detected practically complete loss of BCM in T1DM patients at the same time as it showed hopeful results detecting slight BCM changes over longer periods of time in T2DM patients [262,263,264,265]. Regarding the type of radiomarkers used, recent literature has proposed the usage of the tracer radiomanganese as the first choice in -cell physiology studies [266,267]. Then, this research yielded promising results in an induced T1DM mouse model, in which radiomanganese was captured in the diabetic pancreas in contrast to healthy controls. Later, these findings were also supported by histological quantification of BCM, suggesting that radiomanganese could be considered a subtle new device for the non-invasive evaluation of functional BCM. Regarding other tracers, previous literature proposed the use of monoamine transporter 2 (VMAT2), whose expression has been found to be raised in pancreatic islets compared to exocrine tissue. It was also pointed out how it may be co-expressed with -cells, bringing to light its association with diabetes [268,269,270]. In this case, the ligand used to detect PET radiation is C-dyhidrotetrabenazine (C-DTBZ), due to the affinity that C-DTBZ presents to VMAT2, which has been extensively used in order to evaluate BCM both in humans and mouse models [271,272,273]. According to these findings, it could be considered that PET is an interesting technique that may enhance diabetes comprehension.

Regarding magnetic resonance imaging (MRI), it has been used to examine the role of adipose tissue in energy homeostasis management. In this line, it is necessary to distinguish between fat term and adipose tissue (AT). Hence, fat is primarily a compound of lipids, and more specifically, it is constituted of fatty acids and triglycerides. Moreover, fat is the main component of white adipose tissue (WAT), where it could be also found in different molecules such as water, proteins and minerals [274,275]. In terms of its distribution, most body fat is deposited in AT, although it is also deposited in different organs, including the liver and skeletal muscle. Furthermore, it is essential to note how previous authors pointed out the importance of AT composition and distribution, as it has been extensively related to different metabolic diseases, including obesity, diabetes, and liver disease, in where AT quantities suffered significant changes regarding its composition and location [276,277,278,279]. Then, increased amounts of visceral AT have been related to raised cardiac risk, T2DM, liver disease and cancer [280,281,282,283,284,285,286]. Moreover, while increased liver fat levels have been associated with a higher risk for liver disease and T2DM development [287], as elevated muscle fat levels have been related to raised insulin resistance risk, T2DM risk and decreased mobility [287]. Thus, MRI bases its procedure in analyse different body structures attending their composition, generating images of soft tissues. More specifically, it employs the magnetic properties of the hydrogen nuclei present in water and fat [288,289,290]. Besides, MRI also has been used in order to quantify brown adipose tissue (BAT). BAT has been suggested as a promising target for T1DM and obesity treatment [290], due to its key activity in thermogenesis, obtaining heat by transforming chemical energy [291]. Then, whereas previous literature quantified BAT levels using PET combined with computed tomography (CT) by analyzing BAT depots, located in the cervical-supraclavicular region [292], recent literature proposed more accurate results by using MRI [289]. Furthermore, recent literature suggested the possibility of distinguishing between WAT from BAT, by using quantitative fat-water separated MRI [293]. It would be explained by the fact that BAT contents lower amounts of fat and water, comparing to WAT [294,295]. Then, recent research developed in 2016 suggested interesting results in BAT identifying through MRI in adults. Hence, Giggord and colleagues characterized active and inactive BAT, proposing how MRI would be able to differentiate BAT without human subjectivity without using ionizing radiation, which may enhance understanding of BAT utilities also in difficult populations, including paediatrics, even if the tissue is not activated [294].

## 12. Cell-Based Therapies in Metabolic Diseases

Cell-based therapies may be considered promising methods for the treatment of metabolic diseases. Recent literature proposes the use of these cell-based therapies as an alternative to classical treatments due to their multiple advantages. As an example, the latest literature proposes the use of β—cells transplanted from stem cells as a potential tool to restore insulin secretion in T1DM patients. In the same way, it has been recently suggested that adipose tissue-derived stem cell transplantation may be considered an interesting tool in order to improve glucose metabolism in animal models of obesity and T2DM [295,296,297].

Regarding beta cell transplantation, it has been successfully related to T1DM treatment. As it is well known, T1DM is an autoimmune disease in which T lymphocytes destroy pancreatic beta cells, promoting beta cell islet damage, and consequently blocking insulin secretion, enhancing hyperglycemia. As a result, T1DM is usually associated with several micro- and macrovascular complications, compromising general health status [298]. For many years, T1DM treatment consisted of exogenous insulin administration in order to re-establish insulin levels and allow glucose uptake from cells. Nevertheless, insulin exchange therapy only complements the missing insulin, whereas it does not restore normal pancreatic functioning [299]. Currently, several recent studies have proposed the utilization of beta cell stem cells, including mesenchymal stem cells (MSCs) [300,301], human embryonic stem cells (hESCs) [302,303,304], and bone marrow hematopoietic stem cells (BM-HSCs) [300], which showed encouraging results in restoring immunotolerance as well as preserving islet beta cell function in T1DM patients. Firstly, it is crucial to define stem cells. Stem cells are those that suffer from an undifferentiated state, and they are capable of auto-renewal, initiating nearly any type of tissue or structure [305,306,307,308,309,310]. In general, stem cells have the capacity to enhance the differentiation of islets into beta-cell-like organoids, increasing islet mass, as well as decrease immune responses by reducing transforming growth factor (TGF-) activity and pro-inflammatory processes of T cells and type 1 helper T cells (Th1) [311,312]. More specifically, in terms of mesenchymal stem cells (MSCs), they have demonstrated a protective effect in beta cells as they have immunomodulatory properties. Hence, recent literature suggests that MSCs may inhibit T-cell proliferation, and T1DM MSC-treated patients showed an increase in T regulatory cells, suggesting their autoimmune role [301]. Moreover, the latest literature proposed how MSCs might defeat Th1 progress and postpone T1DM beginning in mice models. Thus, it may be explained through the immunomodulatory effect of MSCs, as lower CD4+ cells were found in mice islets treated with MSCs, as well as a significant decrease in IL-12, IFN-g, p70 tumor necrosis factor, and tumor necrosis factor (TNF) levels [313]. Additionally, apart from their immune activity, MSCs have also shown the ability to increase islet mass, as MSCs quickly undergo mesodermal line differentiation to beta-cell-like cells, subsequently in vitro promoting their transdifferentiation into insulin-producing cells [314]. As a result, recent authors highlighted how these induced beta cell-like cells may stimulate transcription and excretion of insulin due to the fact that they present similar physiological and morphological characteristics to pancreatic islet cells. Hence, in mouse models, it has been suggested that MSCs’ use may successfully control glucose blood levels in diabetic rats [315,316,317]. Apart from mouse models, the newest literature describes how the administration of allogeneic umbilical cord—MSCs in T1DM patients may properly preserve islet beta cells during the first year after being diagnosed, compared to standard treatment [318]. Regarding human embryonic stem cells (hESCs), recent literature has proposed how, in animal models, they may have positive effects in immunodeficient mice and immunocompetent mice and dogs, as hESCs may sustain the survival of human stem cell-beta cells [319]. Additionally, hESCs would also have a positive effect on pancreatic tissue, as they could raise cell proliferation and pancreatic differentiation through TGF-β signaling, as well as promote insulin secretion and improve immunoisolation [320]. Finally, in human models, recent studies showed how pancreatic endoderm derived from hESCs would produce functional insulin-producing cells in vivo, as they improve the survival of pancreatic progenitor cells by decreasing hypoxia and apoptosis.

In adipose tissue-derived stem cell transplantation, new therapies have proposed encouraging results in animal models, as adipose-derived mesenchymal stem cell transplantation may enhance hyperglycemia control through modulating glucose metabolism in the liver of T2DM rats [321]. Hence, adipose tissue-derived stem cells (ADMSCs) present the classic characteristics of MSCs, and consequently, after stimulation, they may suffer differentiation processes, triggering mesodermal and non-mesodermal cell types [322]. Their therapeutic effects are numerous and, in general, constitute a complex process in which immunomodulation, chemoattraction, apoptosis limitation, and local angiogenesis stimulation may occur. All these processes are mediated through a large amount of paracrine factors, involving cytokines, antioxidant substances, and growth factors, which play an important role in different molecular events [323]. Currently, ADMSC-based clinical trials have become an interesting alternative in the management of different metabolic diseases since they comprise the utilization of molecules that present a raised abundance, ease of isolation, rapid growth, and elevated proliferation ability, and they do not present ethical issues [322]. Although the use of BM-MSCS was previously described in the last decade [324,325], recent literature has proposed various advantages that present ADMSCs against BM-MSCS, which may explain the increased use of ADMSCs in the last few years. Thus, recent literature suggested how ADMSCs are robust immunomodulators and offer a better response in oxidative stress and hypoxia-induced adaption, as well as an improved angiogenic force than BM-MSCs [321]. These findings may be explained due to the greater amount of pro-inflammatory and anti-inflammatory cytokines, including IL-6, IL-8, interferon γ (IFN-γ), and TGF-β secreted by ADMSCs, compared to BM-MSCc. Additionally, ADMSCs liberate higher amount of different growth factors, where it could be found in granulocyte colony-stimulating factor (G-CSF), granulocyte macrophage colony-stimulating factor (GM-CSF), nerve growth factor (NGF), or insulin-like growth factor 1 (IGF-1) than BM-MSCc [326,327,328,329]. Thus, recent literature proposed how ADMSCs may have a positive impact both in T2DM and obesity. Regarding T2DM, it has been described how ADMSCs may improve insulin resistance, as they may activate different signaling pathways, including GLUT-4 signaling and PPAR-γ activation, as they may decrease pro-inflammatory cytokines and insulin desensitizing adipokines secretion. Additionally, they could promote insulin production, as ADMSCs may constitute an effective tool in islet beta cells restoring, promoting islet vascularization as well as reducing molecular pathways, such as apoptosis and inflammation. Besides, they have shown the ability to protect endogenous pancreatic islet cells and they have shown the capability to differentiate into insulin-producing cells. Finally, they have demonstrated their high impact on the regulation of hepatic metabolism, since they may decrease gluconeogenesis and reduce hepatic oxidative stress, as well as they have been linked to endogenous hepatocytes regeneration [330,331,332]. Concerning obesity, ADMSCs have shown their potential activity treating atherosclerosis, since they reveal pro-angiogenic and anti-atherosclerotic activities, including endothelial cell growth enhancement and survival, through modulating vascular endothelial growth factor (VEGF), fibroblast growth factor (FGF) and IGF-1 levels. Additionally, they increase angiogenesis as well as promote vascularization, at the same time that they reduce and stabilize the atherosclerosis plaque. Furthermore, ADMSCs also have been related to serum lipid profile improvement, since they decrease total cholesterol, triglycerides and LDL-Cholesterol levels [333,334].

## 13. Practical Applications

The following are practical applications that can be implemented to improve intervention in metabolic diseases:

Multidisciplinary Approach: Adopting a multidisciplinary approach involving healthcare professionals from various fields, such as endocrinology, nutrition, psychology, and exercise physiology, can enhance the effectiveness of interventions. Collaborative efforts allow for the comprehensive assessment and management of metabolic diseases, addressing the complex interplay of physiological, psychological, and behavioral factors [335,336,337,338].

- Patient Education and Support: Providing educational resources and support to patients is crucial in empowering them to manage their metabolic diseases effectively. This includes educating patients about their condition, treatment options, lifestyle modifications, and the importance of medication adherence. Patient support groups and counseling services can also play a significant role in providing emotional support and practical guidance [339,340,341].
- Telemedicine and Remote Monitoring: Embracing telemedicine and remote monitoring technologies can improve accessibility and continuity of care for individuals with metabolic diseases. Remote consultations, mobile applications, and wearable devices enable healthcare providers to monitor patients’ progress, provide real-time feedback, and adjust treatment plans accordingly, even from a distance [342,343,344].
- Behavioral Interventions: Implementing behavioral interventions, such as cognitive-behavioral therapy, motivational interviewing, and mindfulness-based techniques, can help individuals with metabolic diseases make sustainable lifestyle changes. These interventions focus on addressing psychological barriers, promoting self-efficacy, and facilitating behavior modification to support long-term adherence to healthy habits. Early Detection and Risk Assessment: The insights gained from the research on metabolic diseases can be applied in the development of screening tools and risk assessment strategies. By identifying individuals who are at a higher risk of developing metabolic diseases, healthcare professionals can intervene early with targeted interventions, such as lifestyle modifications, to prevent or delay the onset of these conditions [345].
- Personalized Treatment Approaches: The understanding of genetic, psychological, and behavioral factors in metabolic diseases can inform personalized treatment plans. Healthcare providers can consider an individual’s genetic predisposition, psychological factors such as stress and emotional eating, and behavioral patterns to tailor interventions that address the specific needs and challenges of each patient [335,345].
- Nutritional Interventions: The role of nutrition in the development and management of metabolic diseases is crucial. The findings from research can guide the development of evidence-based dietary guidelines and interventions. These interventions can promote healthier eating habits, such as reducing the consumption of processed foods high in fats, sugars, and salt, and increasing the intake of nutrient-dense foods like fruits, vegetables, whole grains, and lean proteins [29,346].
- Exercise Prescription: Prescribing personalized exercise programs, considering factors such as fitness level, health status, and personal preferences, can promote physical activity and improve metabolic health. Collaborating with exercise physiologists or certified fitness professionals can help in designing safe and effective exercise routines and providing ongoing support and guidance [347,348].
- Lifestyle Modification Programs: Physical activity is closely linked to metabolic health. Practical applications derived from research can inform the design of lifestyle modification programs that encourage regular exercise and physical activity. These programs can be tailored to different age groups, fitness levels, and individual preferences, and can include a combination of aerobic exercise, strength training, and flexibility exercises [349].
- Integrative Approaches: Incorporating emerging technologies and therapies can enhance the diagnosis and treatment of metabolic diseases. For example, single-cell transcriptomics can provide insights into cellular-level dysregulation, allowing for targeted therapeutic approaches. Gut microbiota analysis can inform interventions targeting the gut-brain axis and metabolic health. Additionally, advanced imaging techniques can aid in the early detection and monitoring of metabolic diseases [350,351].
- Patient Education and Empowerment: Practical applications of research can be used to develop educational materials and resources for patients with metabolic diseases. Empowering patients with knowledge about their condition, its underlying mechanisms, and the importance of lifestyle modifications can enhance their motivation and adherence to treatment plans [352].
- Long-Term Monitoring and Follow-Up: Regular monitoring of metabolic parameters, such as blood glucose levels, lipid profiles, and body composition, is crucial for evaluating treatment effectiveness and identifying potential complications. Implementing regular follow-up appointments and utilizing digital tools for remote monitoring can facilitate ongoing assessment and timely intervention [335].

Overall, the practical applications derived from the research on metabolic diseases can guide healthcare professionals in implementing preventive strategies, developing personalized treatment plans, promoting healthy lifestyles, and integrating cutting-edge technologies and therapies. By translating research findings into practical applications, we can strive for better outcomes in the diagnosis, management, and prevention of metabolic diseases.

## 14. Limitations and Challenges

Although this review provides a deep insight into new scientific contributions to metabolic diseases, we believe that some of the limitations are as follows:

- Due to the wide diversity of physical exercise, cognitive, and nutritional protocols, it has been difficult to select studies that favor each of the metabolic diseases in particular.
- In diseases as complex and multifactorial as metabolic diseases, establishing general action guidelines is difficult. The connection between psychological and behavioral factors, nutrition, and metabolic diseases demonstrates the need for an integrative approach to disease prevention and treatment.
- The genetic and epigenetic basis of metabolic diseases is still not fully understood, and interventional therapies are still being thoroughly researched.
- Single-cell transcriptomics is an emerging and promising discipline of molecular biology that has the potential to provide unmatched insight into metabolic diseases; however, there are still few studies on the subject, and more information is needed for a full understanding of the processes.
- To assess the long-term stability of microbiota donor engraftment in humans and its associated phenotypes, further studies with larger sample sizes and durations are needed, and the provision of references should be improved in the future.

## 15. Conclusions

This in-depth analysis has shed light on recent discoveries and developments in the diagnosis and treatment of metabolic illnesses, leading to a more thorough comprehension of these conditions. Due to the complex interplay of genetic, psychological, and behavioral variables in the development of metabolic illnesses, a multifaceted strategy is required for effective intervention. The importance of proper diet, exercise, and overall physical activity in preventing and treating metabolic disorders has been emphasized. Single-cell transcriptomics, gut microbiota research, epigenetics, state-of-the-art imaging, and cell-based therapeutics are just a few of the promising new technologies that are being used to better understand and treat metabolic illnesses (Table 1).

Concretely, diabetes mellitus type 1 treatment has been effectively linked to beta cell transplantation. Additionally, it has been shown that PET and MRI are useful tools for enhancing understanding of the pathophysiology of endocrine organs as well as the metabolic pathways regulating glucose and lipid metabolism. Regarding epigenetic medications, such as inhibitors of DNA methyltransferases and histone deacetylases, are presently in development for the treatment of metabolic diseases.

Regarding the psychological profile, it is crucial to analyze the different factors involved in the onset, development, and maintenance of the disease, with special attention to the experiential avoidance process based on junk food, which can become a vicious cycle leading to metabolic damage. Concerning physical activity, studies suggest that supervised training programs are more effective than unsupervised ones. In particular, combined strength and endurance or high-intensity exercise programs show the greatest patient improvements. In terms of nutrition, low-fat diets, and in particular the Mediterranean diet, show the greatest improvements in subjects affected by metabolic diseases. In this regard, understanding these complex relationships between the gut microbiome and types of diets could pave the way for new therapeutic approaches targeting the gut microbiome to prevent or treat metabolic diseases.

The practical applications derived from this review offer tangible approaches for healthcare professionals to enhance their interventions. By implementing a multidisciplinary approach, incorporating personalized nutrition and exercise plans, utilizing telemedicine for remote monitoring and support, and integrating behavioral interventions, healthcare providers can optimize patient care and contribute to the prevention and management of metabolic diseases.

It is crucial to continue research in this field and further explore the practical applications discussed in this review. By translating these insights into clinical practice and public health initiatives, we can make significant strides in reducing the burden of metabolic diseases and improving the overall health and well-being of individuals affected by these conditions. Through ongoing collaboration and innovation, we can pave the way for more effective strategies to combat metabolic diseases and promote healthier lifestyles.

## Figures and Tables

**Table 1:** Sumaryze of principal studies and sections.

Authors and Year	Study Title	Aim of Study	Main Outcomes	Section
**Han et al.** **[60]**	Gene Therapy for Metabolic Diseases	Asses viral-mediated gene addition approaches	Most of the current success has been achieved using a viral-mediated gene addition approach	**Genetic basis**
**Litwin et al.** **[81]**	Negative Emotions and Emotional Eating: The Mediating Role of Experiential Avoidance	Examine whether experiential avoidance would mediate the association between negative emotions and emotional eating	Experiential avoidance mediated the relationship between negative emotions and emotional eating	**Psychological and behavioral basis**
**Hansen et al.** **[100]**	Impact of Endurance Exercise Training in the Fasted State on Muscle Biochemistry and Metabolism in Healthy Subjects: Can These Effects Be of Particular Clinical Benefit to Type 2 Diabetes Mellitus and Insulin-Resistant Patients?	Describe the impact of endurance exercise (training) in the fasted versus fed state on parameters of muscle biochemistry and metabolism linked to glycemic control or insulin sensitivity in healthy subjects	Promising results of exercise (training) in the fasted state have been found in healthy subjects on parameters of muscle biochemistry and metabolism linked to insulin sensitivity and glycemic control	**Physical Activity**
**Ramallal et al.** **[132]**	Inflammatory Potential of Diet, Weight Gain, and Incidence of Overweight/Obesity: The SUN Cohort	Assess the association of the inflammatory potential of a diet using the dietary inflammatory index (DII) with average yearly weight changes and incident overweight/obesity.	Consistently, increases in average yearly weight gains were significantly associated with proinflammatory diets	**Nutrition**
**Hovratin et al.** **[165]**	Toward Modeling Metabolic State from Single-Cell Transcriptomics	Summarize the current state of single-cell metabolic measurement and modeling approaches, motivating the use of computational techniques	Single-cell metabolic modeling is a rising field that provides a new perspective for understanding cellular functions	Single-cell transcriptomics
**Bao et al.** **[178]**	Pseudotime Ordering Single-Cell Transcriptomic of β Cells Pancreatic Islets in Health and Type 2 Diabetes	Evaluate that human pancreatic β cells are heterogeneous and demonstrated the transcript alterations of β cell subpopulation in diabetes.	identified three major states including (1) Normal branch, (2) Obesity-like branch and (3) T2D-like branch based on biomarker genes and genes that give rise to bifurcation in the trajectory. β cell function-maintain-related genes, insulin expression-related genes, and T2D-related genes enriched in three branches, respectively	Single-cell transcriptomics
**Sankararaman et al.** **[184]**	Gut Microbiome and Its Impact on Obesity and Obesity-Related Disorders	Review the current literature on gut microbiome and its impact on obesity and obesity-related disorders	An altered gut microbial composition in obesity and obesity-related disorders is associated with enhanced energy extraction from the non-digestible dietary carbohydrates, increased gut permeability, and increased production of proinflammatory metabolites, such as lipopolysaccharides, resulting in systemic inflammation and insulin resistance	Gut microbiome
**Rönn et al.** **[226]**	A Six Months Exercise Intervention Influences the Genome-Wide DNA Methylation Pattern in Human Adipose Tissue	Describe the genome-wide pattern of DNA methylation in human adipose tissue from 23 healthy men, with a previous low level of physical activity, before and after a six months exercise intervention	A simultaneous change in mRNA expression was seen for 6 of those genes. To understand if genes that exhibit differential DNA methylation and mRNA expression in human adipose tissue in vivo affect adipocyte metabolism	Epigenetics
**Berg et al.** **[248]**	Innovations in Instrumentation for Positron Emission Tomography	Review the current state of the art in PET instrumentation, detectors and systems, describe the major limitations in PET as currently practiced, and offer our own personal insights into some of the recent and emerging technological innovations that we believe will impact the field	Combining these emerging technological and methodological advances promises to lead to a generation of PET scanners tailored for specific applications that really can claim to approach the fundamental limits set forth by the physics of radioactive decay and the statistics of the available signal.	Imaging techniques
**Szot et al.** **[302]**	Tolerance Induction and Reversal of Diabetes in Mice Transplanted with Human Embryonic Stem Cell-Derived Pancreatic Endoderm	Demonstrate that immunotherapies that target T cell costimulatory pathways block the rejection of xenogeneic human embryonic-stem-cell-derived pancreatic endoderm (hESC-PE) in mice	Costimulation blockade prevents rejection of xenogeneic hESC-derived islets; Short-term treatment induces long-term tolerance to xenogeneic hESC-derived islets; Tolerance induced by costimulation blockade is transferable independently of Tregs; Costimulation blockade prevents rejection of allogeneic; hESC islets by human PBMCs	Cell-based therapies

## Data Availability

Not applicable.
